# The Impact of Distance Learning and COVID-19 Lockdown on Students’ Physical Activity and Musculoskeletal Health

**DOI:** 10.7759/cureus.34764

**Published:** 2023-02-08

**Authors:** Konstantina Papageorgiou, Vasileios Mitrousias, Daniil Tsirelis, Georgia Tzika, Alexandros Tsekouras, Nikolaos Zygas, Aristeidis H Zibis

**Affiliations:** 1 Department of Anatomy, School of Health Sciences, University of Thessaly, Larissa, GRC

**Keywords:** covid-19, online learning, sporting activity, musculoskeletal health, distance learning, covid-19 pandemic

## Abstract

Purpose: During the past two years, in most institutions worldwide, educational activities were remodeled for remote delivery, due to the COVID-19 pandemic. The purpose of this study is to assess the effects of two-year distance learning on the physical activity and musculoskeletal health of university students.

Methods: This was a national, cross-sectional study using data collected via an online questionnaire distributed through university communication platforms, which included questions on online education routines, musculoskeletal health, and physical activity of university students.

Results: In total, 1,366 students (65% female, 35% male) from 11 universities took part in the survey. The most common sites of reported pain were the neck (59.5%), shoulders (22.8%), back (29%), and low back (66.7%). Musculoskeletal pain significantly increased during the lockdown, according to the visual analog scale (VAS) for pain (before: 2.7 ± 1.6; during: 5.5 ± 2.2, p<0.001). Everyday pain was referred by significantly more students during the lockdown (4.5% vs 36.1 %, p<0.001), while the percentage of asymptomatic students was significantly decreased (40.5% vs 6.1%, p<0.001). Concerning physical activity, the percentage of students who did not exercise significantly increased during the lockdown (15.1% vs 23.2%, p<0.001). Distance learning and total screen time were positively correlated with VAS for pain scores. On the contrary, an increased frequency of ergonomic position, walking intervals, and physical activity was associated with significantly decreased VAS for pain scores.

Conclusions: Distance learning and limited physical activity led to a significant increase in musculoskeletal pain in university students, while exercise and ergonomic body position were considered protective factors. Interventions to encourage physical activity and healthy studying habits should be developed by universities, since distance learning may be again necessary for the future.

## Introduction

The December of 2019 in Wuhan is considered to be the beginning of a worldwide phenomenon that has brought tremendous changes in daily life. The SARS-CoV-2, responsible for the coronavirus disease 2019 (COVID-19), obligated countries all over the world to proceed with strict measures against the uncontrolled spread of the new, unknown virus. Since March 11, 2020 date on which the World Health Organization (WHO) declared COVID-19 as a pandemic, public health security plans such as social distancing, wearing anti-infection masks, and lockdown restrictions have become part of student’s lives. Since Greece was one of the first countries which followed those measures, the government announced a national lockdown on March 23, 2020, which eventually lasted until May 4, 2020. The second lockdown was announced the following autumn and lasted from November 7, 2020, to May 14, 2021.

During this period, schools and universities shifted to virtual learning alternatives. Furthermore, outdoor activities were permitted only on an individual level, for limited hours daily, and after written confirmation. As a result, physical activity was restricted, and this could have various effects on students’ health. Other studies conducted during this period also report modifications in physical activity. However, contradictory results have been reported [1-4]. In two studies conducted in Italian universities, physically inactive students increased during the lockdown, and this decreased physical activity was associated with increased sitting time and neck pain [1,5]. In fact, this physical inactivity may also be accompanied by inadequate dietary intake and high alcohol consumption, thus leading to a sedentary way of life [6]. On the other hand, in a study conducted on UK students, an increase in the number of steps walked per week was observed from the beginning of the lockdown onwards [3]. Moreover, in a study coming from Spain, an increase in the frequency of physical activity was identified and associated with a reduction in the prevalence of musculoskeletal pain [7]. Interestingly, it seems that pre-COVID activity may affect lockdown behavior and, in a study conducted in France [2] it was reported that initially, active adolescents decreased their physical activity more than those initially inactive.

Taking into consideration these results, the current study was designed to assess the effects of distance learning and lockdown on the physical activity and musculoskeletal symptoms of university students in Greece. This is the first study to detail the impact of the two-year online education and the two lockdowns on students’ musculoskeletal health at a national level. The primary hypothesis of this study is that musculoskeletal pain was increased during distance learning periods. The secondary hypothesis is that maintenance of an active lifestyle and healthy studying habits are protective factors for musculoskeletal symptoms.

This article was previously presented as a meeting abstract at the 2022 AMEE annual conference on August 30, 2022. It was also previously posted on the Research Square preprint server on October 28, 2022.

## Materials and methods

Launch of the study

In June 2021, an online questionnaire prepared by a team of four medical students, in cooperation with two orthopedic surgeons was released. The questionnaire was distributed to students from several Greek universities through university communication platforms. All participants were informed about the purpose of the study and the security of their anonymity and their personal data, according to the Greek and European legislation before replying. All students were encouraged to answer with honesty and as further motivation for participating and finishing the questionnaire, a home exercise guide that aimed to relieve common symptoms of low back and neck pain was provided after submission of the questionnaire.

**Figure 1:**
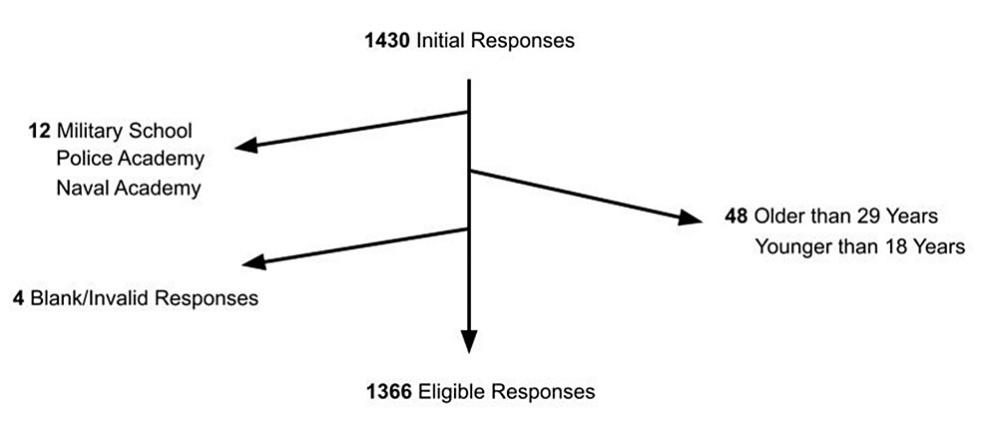
The flowchart of the study.

One month later, 1,430 responses were received out of the 1,826 distributed questionnaires (response rate 78%). Exclusion criteria were age <18 and >29 and students studying in military schools or the police academy. Blank and partially filled questionnaires were also rejected. A flowchart summarizing the above is available in Figure 1.

Structure of the questionnaire

The questionnaire was divided into four sections. The first section covered demographic and physical characteristics questions including sex, age, weight, and height. The second section included questions regarding students’ habits during online education such as the type of device used, hours of attendance, and the existence or not of an ergonomic position. The third section consisted of questions regarding the participants’ musculoskeletal health, namely the intensity and location of pain prior to, during, or after the lockdown and the possible need for consultation. Finally, the fourth section inquired students about the physical activity before and during quarantine. The questionnaire is available in the Appendix.

Data management and statistical analysis

All data were analyzed using the SPSS statistical package, version 21.0 for Windows (https://www.ibm.com/analytics/spss-statistics-software, IBM Corp., Armonk, NY, USA). Descriptive and inferential statistics were performed. The level of statistical significance was set at 0.05.

The paired-samples t-test was used to assess differences in the visual analog scale (VAS) for pain scores before and during the lockdown. The VAS for pain is considered a valid and reliable method of assessing pain and in fact, it seems to be less influenced by non-pain intensity factors [8]. The one proportion Z test was used to assess differences in students’ proportions. A Spearman's rank-order correlation was run to assess the relationship between VAS Pain score (during lockdown) and BMI, distance learning time, total screen time, frequency of walking intervals, frequency of ergonomic position, break time, frequency of physical activity before lockdown and during the lockdown. The monotonic relationship was assessed by visual inspection of a scatter plot.

Institutional ethics statement

The research was conducted in accordance with the principles embodied in the Declaration of Helsinki and in accordance with local statutory requirements. All participants were informed about the purpose of the study and gave written informed consent to participate in it.

## Results

Demographic characteristics of the study population

**Figure 2:**
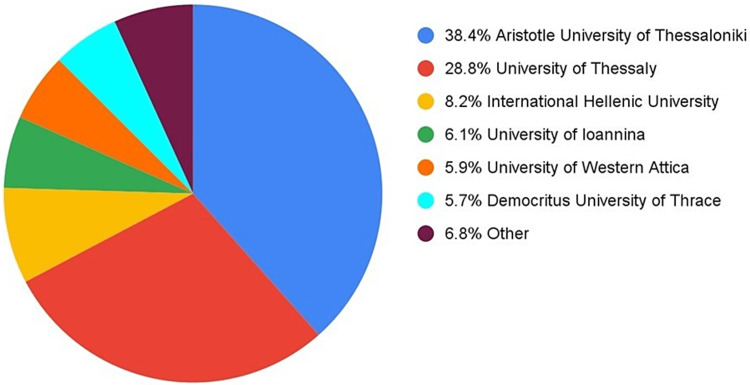
Universities participating in the study.

 In total, 1,366 students were included in the study, 887 (65%) men and 479 (35%) women. The mean age of students was 20.61 years old (18-29), mean BMI was 24.08 for males and 21.97 for female students. As shown in Figure 2, 525 students responded from the Aristotle University of Thessaloniki, 394 from the University of Thessaly, 112 from the International Hellenic University, 84 from the University of Ioannina, 80 from the University of Western Attica, 78 from the Democritus University of Thrace and the rest 93 are other various universities including the University of Crete and the University of the Aegean. Nearly 40% of the respondents (553) were healthcare students.

Online education

The majority of the students (92.6%) took their online courses via Computer (PC or Laptop), while 4.9% used smartphones, 2.4% used tablets and only one participant (0.1%) used Smart TV. Among the participants, 17 (1.2%) attended their online lessons for more than eight hours per day, 7.8% for 6-8 hours, 15.2% for less than two hours, and the largest group of the students averaged 2-4 hours (35,7%) or 4-6 hours per day (40.0%). Moreover, participants also gave information about their daily average screen time. In this question, the percentage of students that answered less than four hours per day was 5.8%, 4-6 hours 20.3%, 6-8 hours 35.7% and 522 students (38.2%) answered more than eight hours. Finally, students were asked about their ergonomic or not position while taking their online courses, rating it from 1 to 5 (never to always). In this section, the mean answer was 2.1 and only 108 participants (7.9%) answered 4 or 5.

Musculoskeletal symptoms

**Figure 3:**
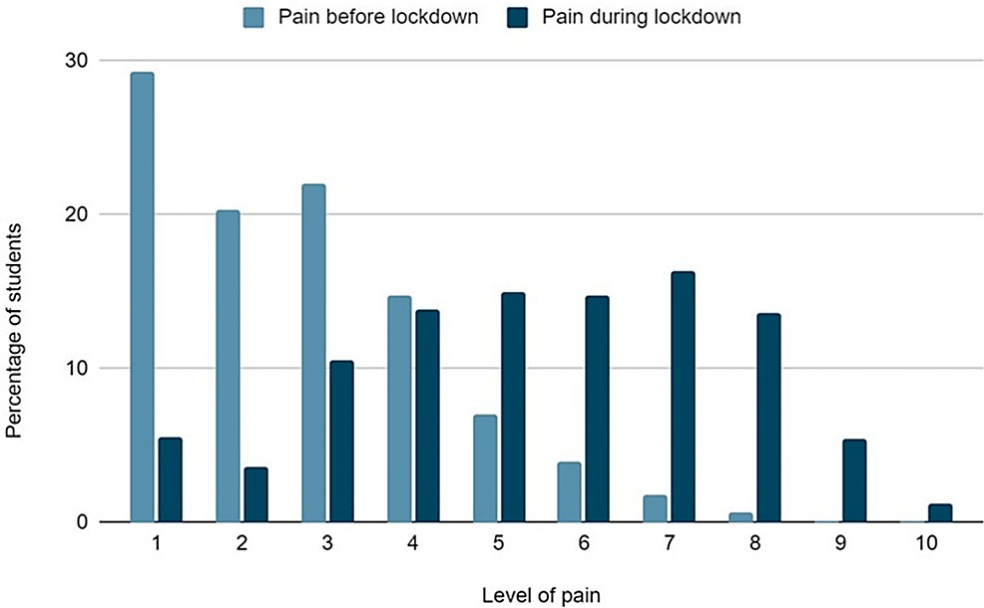
Intensity of pain before and during the lockdown.

**Figure 4:**
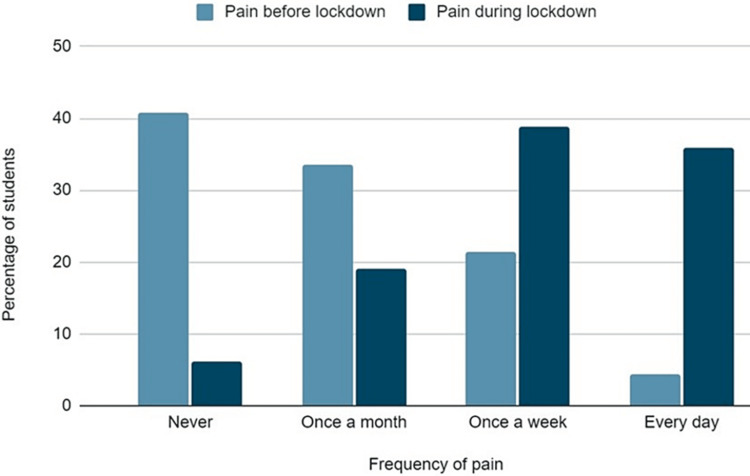
Frequency of pain before and during the lockdown.

The intensity and frequency of musculoskeletal pain is presented in Figures 3, 4, respectively. Pain was significantly increased during the lockdown, as shown by the answers in the VAS pain scale (Mean VAS Score Before lockdown: 2.7 ± 1.6; During lockdown: 5.5 ± 2.2, p<0.001). Everyday pain was referred by significantly more students during lockdown (4.5% vs 36.1 %, p<0.001), while the percentage of asymptomatic students was significantly decreased (40.5% vs 6.1%, p<0.001).

Physical activity

**Figure 5:**
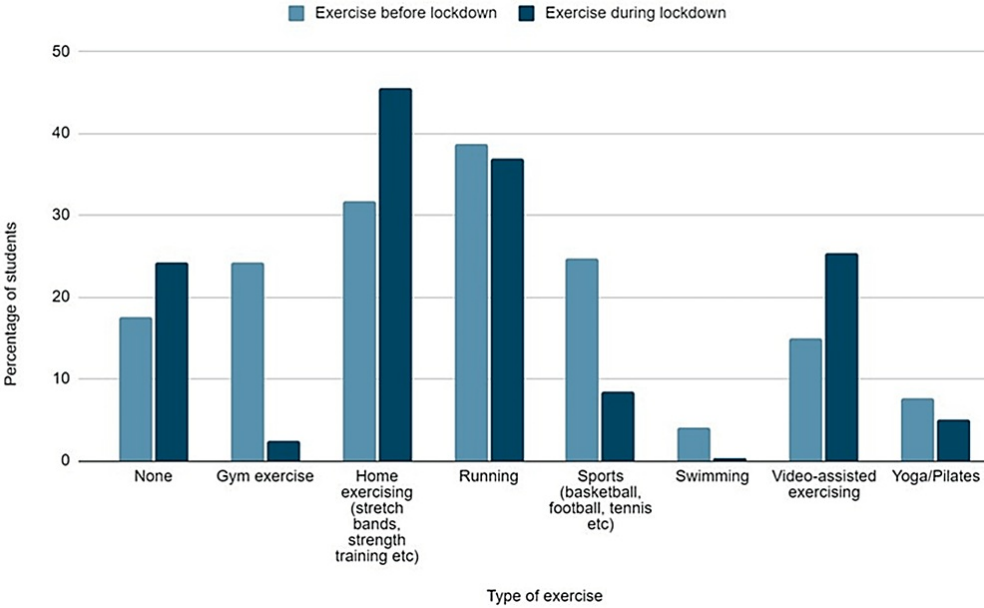
Type of exercise before and during the lockdown.

**Figure 6:**
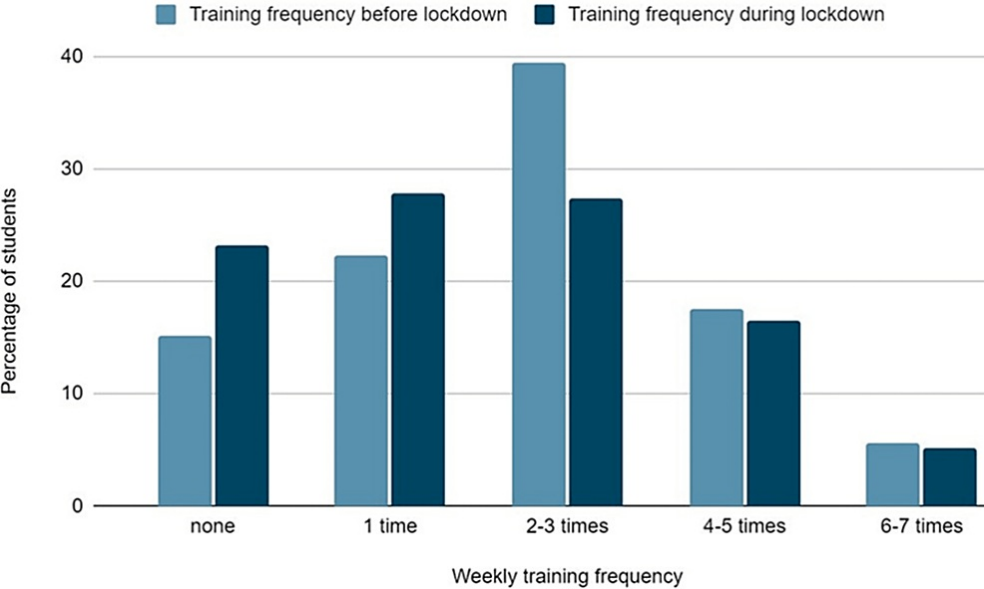
Weekly frequency of exercise before and during the lockdown.

The type and frequency of exercise are presented in Figures 5, 6, respectively. The percentage of students who didn’t exercise at all, significantly increased during the lockdown (15.1% vs 23.2%, p<0.001). The percentage of students exercising at home or practicing video-assisted training programs was significantly increased during lockdown (31.4% and 14.6% prior to lockdown vs 45.1% and 24.8% during lockdown, p<0.001). On the contrary, a statistically significant decrease was observed in gym exercise, team sports, and swimming (24.4%, 24.6%, and 4.3% vs 2,5%, 8.4%, and 0.3%, p<0.001). Moreover, a small decrease was observed in students practicing yoga/pilates (7.6% vs 5.1%, p=0.02). However, no difference was observed in running (38.4% vs 37.3%, p=0.58). The majority of students (46.5%) stated that during the lockdown, they reduced the time devoted to exercise. However, 30.9% stated that they increased the time devoted to exercise.

Correlations

The Spearman's rank-order correlation was run to assess the relationship between VAS pain score (during lockdown) and BMI, distance learning time, total screen time, frequency of ergonomic position, frequency of break time, and frequency of physical activity before lockdown and during the lockdown. Preliminary analysis showed the relationship to be monotonic for all the above variables, as assessed by visual inspection of a scatter plot. Results are summarized in Table 1. Distance learning and total screen time were positively correlated with VAS pain scores. On the contrary, an increased frequency of ergonomic position, walking intervals, and physical activity levels before and during lockdown was associated with significantly decreased VAS pain scores. BMI was shown to also have a negative correlation, although not statistically significant (p=0.396).

**Table 1:** Correlation between VAS for pain score (during lockdown), studying habits, and exercising frequency.

Variable	Correlation Coefficient (rs)	P
BMI	-0.023	0.396
Distance learning time	0.128	<0.005
Total Screen time	0.070	0.01
Ergonomic Position Frequency	-0.133	<0.005
Walking Intervals Frequency	-0.096	<0.005
Physical Activity Before Lockdown Frequency	-0.076	<0.005
Physical Activity After Lockdown Frequency	-0.125	0.005

Differences between male and female students

**Figure 7:**
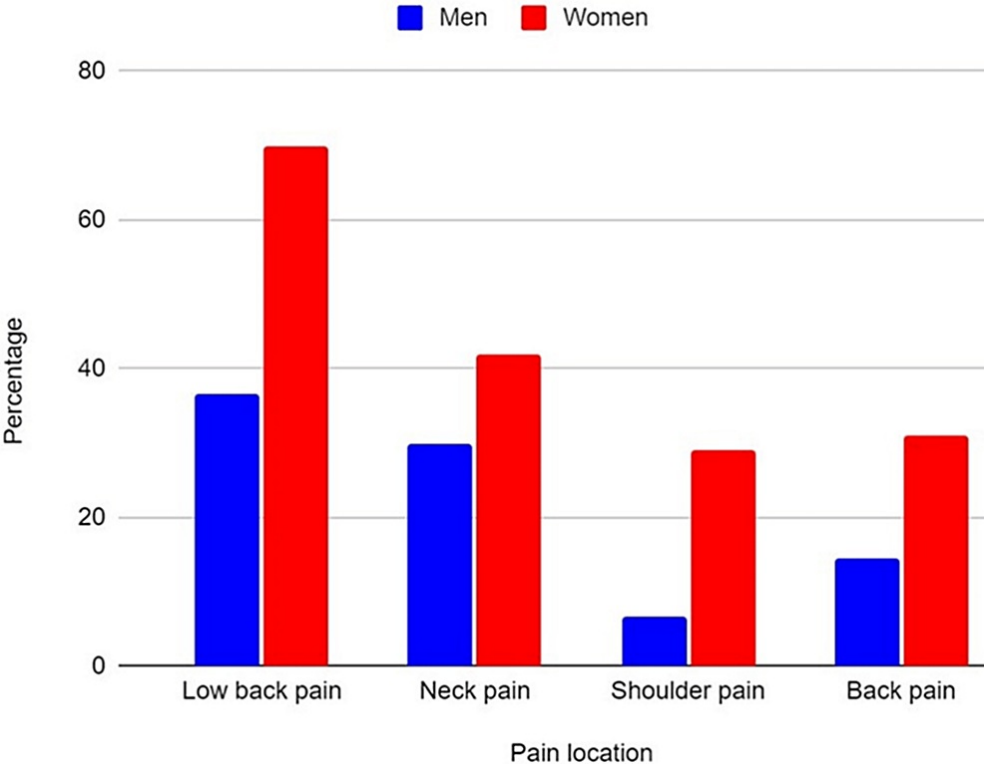
Pain location in male and female students.

As shown in Figure 7, back pain, low back pain, and neck pain were more common in female students compared to male students (31% vs 14.6%, p<0.05; 70% vs 36.7%, p<0.05; 42% vs 29.8%, p<0.05). Shoulder pain was also more common in female students compared to male students (29% vs 6.5%, p<0.05). Statistically, significantly more male students reduced their physical activity during lockdown compared to female students (51% vs 43%, p<0.05), and moreover, more female students increased their physical activity during lockdown compared to male students 34% vs 24%, p<0.05).

## Discussion

University students in Greece have spent more than nine months studying virtually with online learning alternatives. As indicated by our results, everyday pain was referred by significantly more students during the lockdown, while the percentage of asymptomatic students was significantly decreased, which confirms the primary hypothesis of this study This result parallels other studies conducted during and after lockdown [5,9], that also highlight musculoskeletal pain in numerous body locations.

The other worth-mentioning finding of this study is that the increased frequency of ergonomic position and walking intervals before and during lockdown was associated with significantly decreased VAS pain scores, which confirms the secondary hypothesis of the present study. Thus, the need to encourage proper posture awareness among university students should not be neglected. According to a similar study [10], lack of ergonomic position was one of the risk factors combined with a long period of sitting and not enough physical activity for low back pain. In addition to schools, instructional and educational programs for promoting healthy postural behaviors have been implemented in the workplace and other community settings [11]. These programs have been shown to help people become more conscious of their posture and change their habits and their use should be also examined for university students.

Fortunately, the results suggest that musculoskeletal pain caused by distance education didn’t raise so much concern, since only 11.4% of the students believed that their symptoms were severe enough in order to consult a doctor. Furthermore, 57.8% stated that the pain was reduced soon after the end of the remote teaching model which reinforces the idea that links pain to this new lifestyle. Therefore, even for a limited period, the distance learning strategy has prompted an increase in musculoskeletal pain in this specific population.

The above implies that students should be targeted for health promotion interventions aimed at maintaining and improving physical activity and dietary practices. According to the new World Health Organization Report, the ongoing COVID-19 pandemic has highlighted the importance of being physically active as part of daily life. In Greece, new health-enhancing physical activity policies were adopted according to the 2021 Physical Activity Factsheet, although targeted interventions for university students were not included [12]. Health institutions should implement measures that encourage physical activity, for the promotion of good health and improvement of students' quality of life, transitioning after this pandemic and beyond. Other researchers gathered and studied the effectiveness of different strategies for physical activity promotion in the university setting, including but not limited to health promotion courses, physical activity, exercise, or sports programs attendance and pedometers or activity trackers, measures which should be considered even as a permanent intervention [13].

Physical inactivity is considered another pandemic by itself [14]. Before the COVID-19 outbreak, globally, 23% of adults and 81% of adolescents (aged 11-17 years) did not meet the World Health Organization's global recommendations on physical activity for health [12], and the trend was that physical inactivity was not increasing, in contrast to the time spent on sedentary behavior [15]. However, the results of our survey reveal that the majority of students managed to maintain physical exercise, with 76.8% of them exercising at least once per week. The modes of physical activity were limited to running, home-based exercising, and video-assisted exercise, taking into account that the national restrictions did not permit team sports. Interestingly, an increase in physical activity was observed in some participants and this could be explained by easier access to exercise equipment at home or greater support to become more active during home confinement.

Adding to the above, the total screen time in our survey was also positively correlated with VAS pain scores. This fact indicates the need for the development of hybrid teaching approaches in the transitional after-COVID period. A stimulating learning environment comprising physical and virtual learning spaces will support diverse learning needs and enable the adoption of flexible pedagogies. This is the first time that present generations face a global pandemic, disrupting higher education institutes (HEI) and the past two years have certainly been challenging for both academics and students. It is essential to learn about students' daily routine during this period in order to be better prepared for subsequent disruptions to HEI and to understand how COVID-19 has shaped life in and out of the campus, particularly as research has shown the substantial impact of the pandemic on mental health and wellbeing across the general population [16]. Moving forward, it is important to ensure higher education delivery is well-equipped to transition to changing circumstances and future restrictions on students accessing the university campus.

Finally, with regard to the gender differences, the findings of our study indicate that females experienced more pain during lockdown, but they also significantly increased their physical activity. This result aligns with another study [7] which showed that the frequency of physical activity carried out increased significantly during the period of confinement. The reason for this contradictory finding is not entirely clear but it could be interpreted as activity-related pain, which was higher in the more active group, or being a result of a disproportionate ratio of females to males since women accounted for 65% of our sample. It could be also attributed to dysmenorrhea, although this type of pain is usually clearly recognized by females and may only affect the percentage of low-back pain. Another plausible explanation could be that the motivation (peer pressure, weight loss, or maintenance) for physical activity among females led to higher levels of physical activity particularly during university enrollment when body weight is likely to increase. And finally, there is also literature to support that females have increased pain sensitivity compared to males [17].

Limitations

One of the limitations of the present study is the inability to control factors that affect the perception of pain. Each person experiences pain differently, and this difference is associated with the person’s personal background, interpersonal context, and other non-physiological factors such as the type of personality, beliefs, and other socio-cultural variables. Additionally, the validity and reliability of VAS for musculoskeletal disorders have been shown to be satisfactory but not flawless. Moreover, other causes that may have led to limited physical activity such as a COVID-19 infection or a traumatic event have not been investigated. Another limitation is that diet and nutrition were not assessed in this study and finally, musculoskeletal symptoms have been self-reported by students, and not clinically verified.

## Conclusions

Lockdowns imposed as a consequence of the COVID-19 global pandemic led to an increase in musculoskeletal pain in university students since distance learning without an ergonomic position and limited physical activity were adopted during these periods by the majority of students. However, a certain percentage of students maintained or even increased their activity, by modifying their exercise routine in a home-based way (e.g., video-assisted training) or insisting on their running habits. This was mostly observed in female students, which were found to be more active, although these higher levels of activity may have led to an activity-related increase in pain. Since distance learning seems to gain support, especially as an alternative to traditional teaching for specific periods and subjects, universities and educators should focus on the development of interventions to encourage physical activity and healthy studying habits among students.

## Appendices

Online Questionnaire

Section  1 of 4 - Demographic Data

1. Gender

Man

Woman

2. Age

[Fill in] 

3. Weight (in Kg)

[Fill in] 

4. Height (in cm)

[Fill in] 

5. University (e.g. University of Thessaly)

[Fill in]  

6. Department (e.g. Department of Medicine)

[Fill in]  

7. Year of studies (e.g. 3rd)

[Fill in] 

Section 2 of 4 - Distance Learning

1. Which tool do you usually use to attend university classes - study at home?

·        Tablet

·        Smartphone

·        Personal Computer

·        Smart TV

2. How many hours of online courses do you attend per day on average?

·        0-2

·        2-4

·        4-6

·        6-8

·        >8

3. How many hours of total screen time do you have per day (Including time on social media, watching series, etc.)?

·        0-2

·        2-4

·        4-6

·        6-8

·        >8

4. How often do you attend online classes in a specially adapted place (e.g., desk with office chair)?

·        Never

·        1

·        2

·        3

·        4

·        5

·        Always

5. Where else do you attend your classes? (1 = never, 5 = always)

·        Bed: 1-5

·        Sofa: 1-5

·        Dining table: 1-5

6. Do you maintain an ergonomic posture, as shown in the picture?

·        Never

·        1

·        2

·        3

·        4

·        5

·        Always

7. How many hours of online classes do you attend before getting up from the sitting position?

·        < 1

·        1

·        2

·        3

·        >3

Section 3 of 4 - Symptoms Related to the Musculoskeletal System

1. How often were you experiencing pain BEFORE quarantine started, while participating in any type of distance learning?

·        Everyday

·        Once a week

·        Once a month

·        Never

2. How often were you experiencing pain DURING the quarantine period, while participating in any type of distance learning?

·        Everyday

·        Once a week

·        Once a month

·        Never

3. Where was the pain located?

·        Neck (Cervical spine)

·        Thorax (Thoracic spine)

·        Back (Lumbar spine)

·        Shoulders

·        Feet

·        Other

4. How intense was the pain BEFORE quarantine?

·        1 (No pain) to 10 (very intense)

5. How intense was the pain DURING quarantine?

·        1 (No pain) to 10 (very intense)

6. Was a medical appointment necessary for these symptoms?

·        Yes

·        No

7. Did the symptoms subside AFTER quarantine and by the return to normal activities?

·        Yes

·        No

Section 4 of 4 - Physical Activity

1. Frequency of physical activity BEFORE quarantine

·        Never

·        Once a week

·        2-3 times a week

·        4-5 times a week

·        6-7 times a week

2. Type of physical activity BEFORE quarantine

·        None

·        Running

·        Exercise at home

·        Exercises instructed via videos

·        Gym

·        Swimming pool

·        Sports (Basketball, Football, Tennis, etc.)

·        Yoga or Pilates

3. Frequency of physical activity DURING quarantine

·        Never

·        Once a week

·        2-3 times a week

·        4-5 times a week

·        6-7 times a week

4. Type of physical activity DURING quarantine

·        None

·        Running

·        Home workouts

·        Video workouts (any kind of workout)

·        Gym

·        Swimming pool

·        Sports (Basketball, Football, Tennis, etc.)

·        Yoga or Pilates

5. During the quarantine period in total?

·        I increased the duration of exercise

·        I decreased the duration of exercise

·        I did not change the duration of exercise
